# Energy Storage Analysis of a Mixed R161/MOF-5 Nanoparticle Nanofluid Based on Molecular Simulations

**DOI:** 10.3390/ma11050848

**Published:** 2018-05-20

**Authors:** Qiang Wang, Shengli Tang, Leilei Li

**Affiliations:** 1 Key Laboratory of Low-grade Energy Utilization Technology & System, Ministry of Education, College of Power Engineering, Chongqing University, Chongqing 400044, China; wangqiang870609@163.com (Q.W.); tytsl@cqu.edu.cn (S.T.); 2 College of Aerospace Engineering, Chongqing University, Chongqing 400044, China; 3 Key Lab. of Opto-Electronic Technology & System, Ministry of Education, Chongqing University, Chongqing 400044, China

**Keywords:** R161, MOF-5, adsorption, thermal energy storage, molecular simulation

## Abstract

The thermal properties of refrigerants can be modified by adding porous nanoparticles into them. Here, molecular simulations, including molecular dynamics and grand canonical Monte Carlo, were employed to study the thermal energy storage properties of an R161/MOF-5 nanofluid. The results show that the thermodynamic energy change of MOF-5 nanoparticles is linear to the temperature. The adsorption heat calculated by grand canonical Monte Carlo is close to that calculated by the Clausius–Clapeyron equation. Additionally, a negative enhancement of the thermal energy storage capacity of the R161/MOF-5 nanofluid is found near the phase transition area.

## 1. Introduction

With the rapid development of modern society, serious problems, such as energy shortage and environmental pollution, have become increasingly prominent [1,2]. Energy conservation and emissions reduction are effective ways to alleviate the energy and environmental problems. The thermodynamic cycle is the main approach for energy conversion. Additionally, the working fluid is the energy carrier of the thermodynamic cycle. Therefore, using various means to improve the thermophysical properties of working fluid can improve the efficiency of energy conversion.

Dispersing nanoparticles into traditional working fluids, e.g., water, alcohol, oil, and refrigerants, to produce uniform and stable nanofluids can effectively improve the thermal conductivity of working fluids [3,4,5,6]. Nanofluids have great prospects for application in the fields of energy, chemical engineering, automobiles, building and construction, etc. The enhancement of the thermal conductivity of nanofluids is mainly due to the effective medium theory [3]. Also, the layering phenomenon, Brownian motion, clustering, ballistic phonon motion, thermal boundary resistance, and mass difference scattering will impact on the thermal conductivity of nanofluids [7,8,9,10].

Besides this, a nanofluid with porous materials can be used for thermal energy storage [11]. Carbon nanotubes, zeolites, and Metal-Organic Frameworks (MOFs) are common nanoporous materials. These materials have a very large specific surface area compared to conventional materials. Thus, extra energy can be stored and output by the fluid desorbing/adsorbing in the nanoporous materials. Chen et al. [12] performed comprehensive research on the energy storage of carbon nanotube nanofluids under the action of heat, force, and electric coupling. McGrail et al. [13] proposed to use metal-organic heat carrier nanofluids (MOHCs, a refrigerant to which has been added MOF nanoparticles to form the nanofluid) to improve the efficiency of the Organic Rankine cycle (ORC, using refrigerants as the working fluid for the Rankine cycle). They reported [14] noticeable adsorption and reversible desorption of refrigerant molecules in MOFs. Therefore, MOHCs have great potential in low-grade energy utilization and refrigeration cycles.

Theoretically, the heat adsorption energy of MOHCs (h_MOHCs_) is mainly composed of three parts: (i) the enthalpy change of the working fluid (∆h_Fluid_); (ii) the thermodynamic energy change of MOF particles ((∫C_p_dT)_MOFs_); and (iii) the desorption heat of the fluid in MOFs (∆h_desorption_), i.e., [11,13],

∆h_MOHCs_ = (1 − x) ∙ ∆h_Fluid_ + x ∙ (∫C_p_dT)_MOFs_ + x ∙ ∆h_desorption_ (1)

where x is the mass fraction of MOFs in the MOHC. Additionally, Equation (1) can be written as

∆h_MOHCs_ = ∆h_Fluid_ + x ∙ ((∫C_p_dT)_MOFs_ + ∆h_desorption_ − ∆h_Fluid_) (2)

It can be inferred from Equation (2) that MOHCs are able to store more thermal energy than pure fluid when the sum of the thermodynamic energy change of MOF particles and the desorption heat of the fluid in MOFs is larger than the enthalpy change of the working fluid.

**Figure 1:**
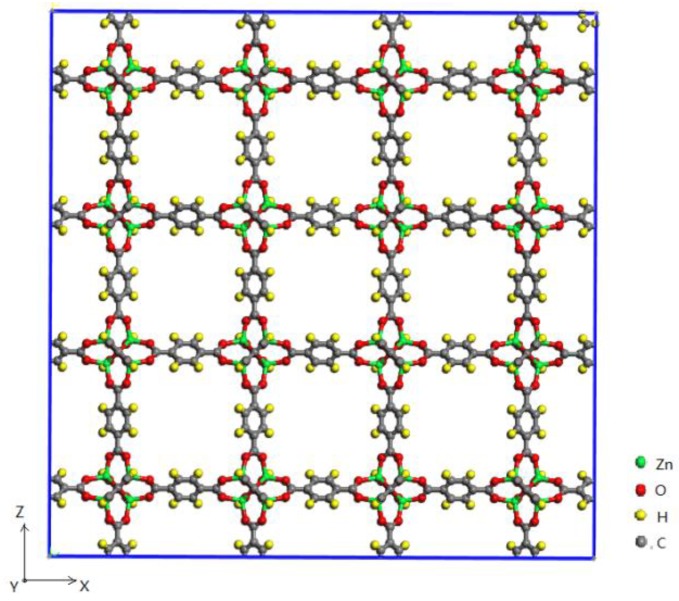
2 × 2 × 2 unit cells of MOF-5.

MOFs are a class of organic–inorganic hybrid materials constructed from organic ligands and inorganic metal units. They own a large specific surface area, large porosity, a diverse structure, and thermal stability, which have provided MOFs with a wide range of applications in the fields of energy, chemicals, materials, and so on [15]. In the numerous MOF structures, MOF-5 [16] is an MOF that consists of Zn_4_O clusters connected by 1,4-benzodicarboxylate linkers as shown in Figure 1. It has a specific surface area of 2000 m^2^/g and a good thermostability whose structure remains stable below 573 K [17]. Fluoroethane, also known as R161, is one of the most used organic refrigerants with zero ozone depression potential, low global warming potential, and good thermal properties. Additionally, the adsorption properties of R161 in MOF-5 have been rarely reported [14,18]. Thus, the thermal energy storage property of an MOHC based on R161 and MOF-5 is investigated in the present paper.

Besides this, due to the length scale of the nanopore structure in MOFs being small, it is difficult to investigate the thermal energy storage mechanism of the organic working fluid in MOFs by using conventional experimental and theoretical methods. Molecular simulation [19] is an ideal tool to reveal the micro-mechanisms of materials. Therefore, molecular simulation is employed to investigate the thermal energy storage of R161/MOF-5-based MOHCs. The work is also expected to provide useful insights on adsorption refrigeration and heat pumps [20].

## 2. Method and Computational Details

According to the thermodynamic principles discussed in Equation (2), the thermodynamic parameters of a pure working fluid are classic, and can be obtained by an experimental or a theoretical method. Since MOFs are a novel material, the thermal properties of MOFs and the adsorption properties of the refrigerant in an MOF need further research. Therefore, here, the thermodynamic parameters of R161 were obtained from the National Institute of Standards and Technology (NIST [21]). The thermodynamic energy change of MOF-5 can be obtained by molecular dynamics (MD) simulation [22]. Additionally, the desorption heat of R161 in MOF-5 can be easily calculated from grand canonical Monte Carlo (GCMC) simulations [23].

### 2.1. Simulation Models

**Figure 2:**
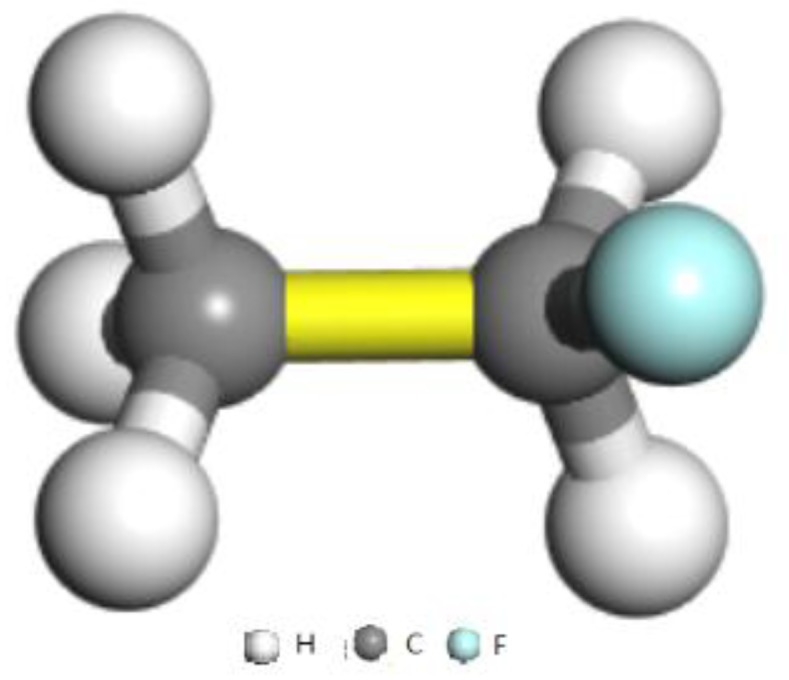
Molecular configuration of R161.

The calculation model of MOF-5 comprising 2 × 2 × 2 unit cells (X: 51.788 Å, Y: 51.788 Å, Z: 51.788 Å) is shown in Figure 1 with 1536 carbon atoms, 832 oxygen atoms, 768 hydrogen atoms, and 256 Zn atoms. The structure of R161 (CH3CH2F) is shown in Figure 2. The MD simulations and GCMC simulations were performed using Materials Studio [24]. In the simulations, the COMPASS force field [25] was used to describe the intra and inter molecular interactions. The Ewald method [26] was used to deal with the long-range Coulombic interactions. Periodic boundary conditions were applied in the X, Y, and Z directions in all of our simulations.

### 2.2. MD Simulation Details

The MD simulations were performed in the Forcite module of Materials Studio. The timestep was set as 1 fs and the system was equilibrated for 100 ps to calculate the thermodynamic energies of MOF-5 nanoparticles at 280, 300, 320, 340, 360, 380, 400, and 420 K. The simulations were computed in the canonical ensemble (NVT) ensemble with the Berendsen method [27] to control the temperature.

### 2.3. GCMC Simulation Details

The GCMC simulations were performed in the Sorption module of Materials Studio. The adsorption isotherms (280, 300, 320, 340, 360, 380, 400, and 420 K) of the adsorption of R161 in MOF-5 nanoparticles were calculated from 0 to 6000 kPa. The fugacity was calculated by the Peng–Robinson equation. For each point of the adsorption isotherms, the equilibration time was 10,000 steps with another 100,000 steps for statistics.

## 3. Results and Discussion

### 3.1. Thermodynamic Energy of MOF-5

**Figure 3:**
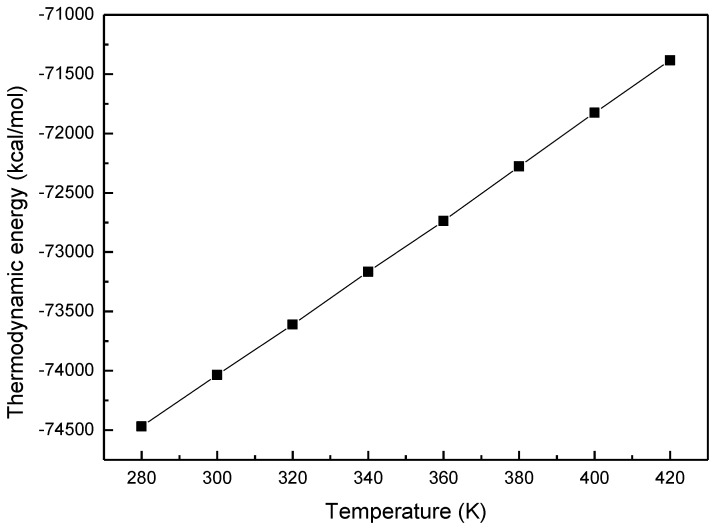
The thermodynamic energy of MOF-5.

The thermodynamic energies of MOF-5 at different temperatures are plotted in Figure 3. It shows that the thermodynamic energy of MOF-5 increases linearly with the temperature rise. The increment of internal energy per unit of temperature is C_p_ of MOF. Here, the ρc_p_ = 1.24(J/cm^3^·K), which is close to the value of a typical MOF, 1.44 J/cm^3^·K [13,28].

### 3.2. Adsorption Isotherms and Enthalpy of Desorption

**Figure 4:**
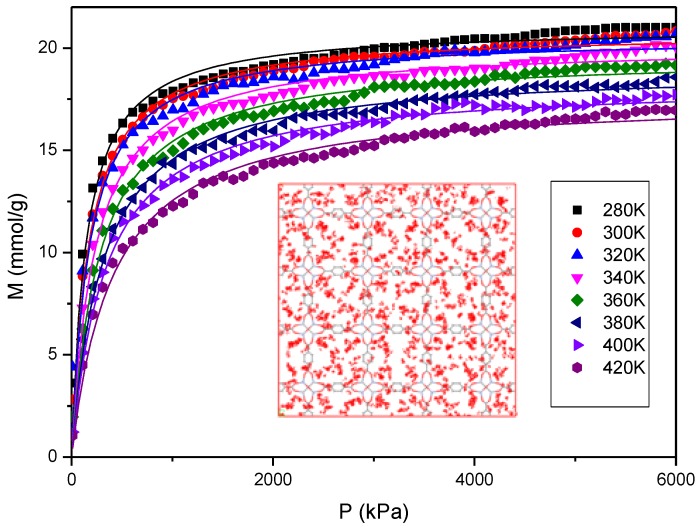
Adsorption isotherms of R161 adsorbed in MOF-5.

The adsorption isotherms of R161 adsorbed in MOF-5 are shown in Figure 4. The adsorption isotherms are also fitted by the Langmuir equation, details of which are shown in Appendix A. The adsorption snapshot in Figure 4 shows that R161 mainly distributes around the O and Zn atoms of MOF-5 because these atoms are more attractive than the benzene ligand. Also, the adsorption isotherms decrease as the temperature rises. This is because the raised temperature results in the increased motion of molecules. Thus, the kinetic energy of the fluid molecules can overcome the adsorption energy of the MOF structure. This is the basis for thermal energy storage in MOHCs.

The enthalpy of desorption can be calculated by the Clausius–Clapeyron (C–C) equation based on the adsorption isotherm,

(3) $${\left(\frac{\delta \ln P}{\delta T}\right)}_{\theta }=\frac{q_{st}^{}}{RT^{2}}$$

It can be rewritten as:

(4) $$\ln P=-\frac{q_{st}}{R}\frac{1}{T}+C$$

**Figure 5:**
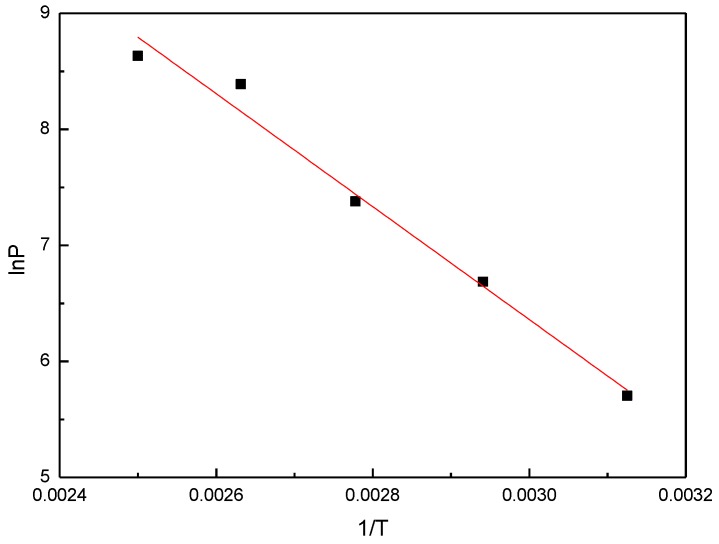
The relationship between lnP and 1/T of R161 adsorption in MOF-5.

Thus, the desorption heat *q_st_* can be obtained by calculating the slope relationship between ln*P* and 1/*T*, which is shown in Figure 5. Additionally, the results of the desorption heat calculated by the C–C equation and GCMC simulations are listed in Table 1. The desorption heat calculated by GCMC simulations is close to that calculated by the C–C equation. With consideration of the calculation error of the C–C equation, the desorption heat calculated by GCMC will be used to calculate the thermal energy storage in the following section.

### 3.3. Thermal Energy Storage

**Figure 6:**
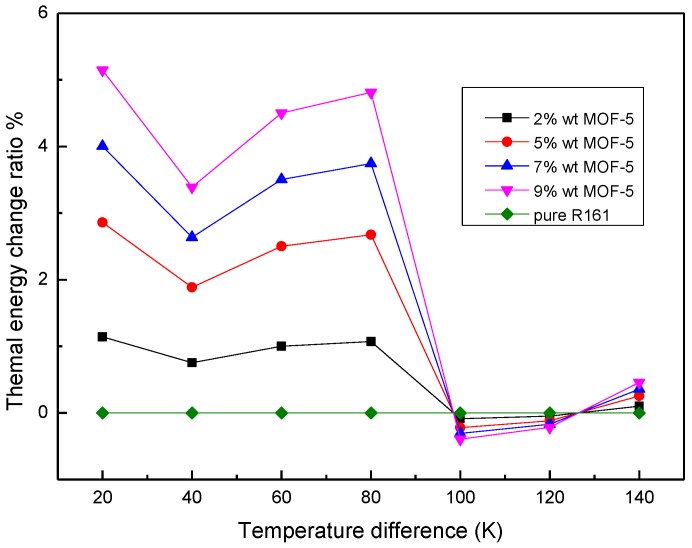
The relative energy change of different working fluids as the temperature difference is increased at 5 MPa.

**Figure 7:**
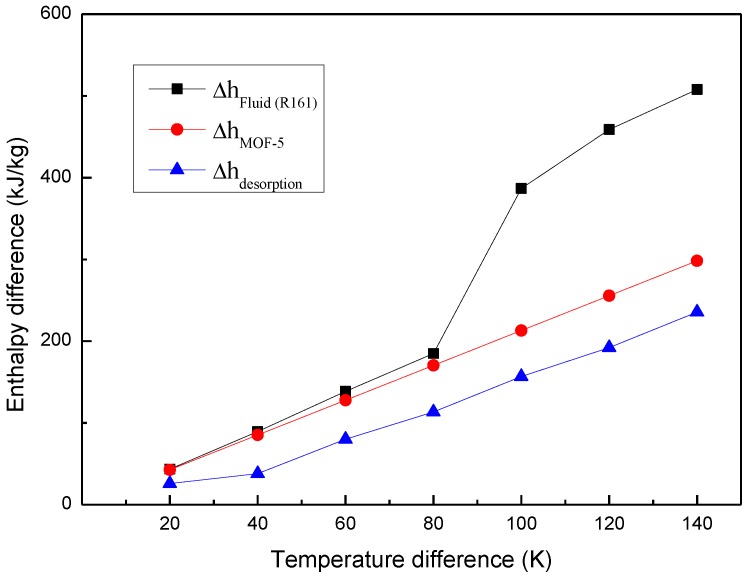
The relationship between enthalpy difference and temperature difference at 5 MPa.

Then, the thermal energy storage capacity of MOHCs can be computed according to Equation (1) or Equation (2). As aforementioned, the enthalpy difference of MOHCs at a different temperature is the thermal energy storage capacity. Thus, the thermal energy storage capacity of MOHCs with a different mass ratio of MOF-5 is presented as Figure 6. The temperature of the cold source was set as 280 K. Here, the enthalpy difference of pure R161 was considered to be the reference working fluid. Also, the thermodynamic energy change of MOF particles and the desorption heat of the fluid in MOFs are calculated and plotted in Figure 7. Note that the R161 experiences a phase transition when the temperature difference is about 100 K. The enthalpy difference of pure R161 experiences a sharp increase near this temperature range. The thermodynamic energy change of MOF particles and the desorption heat of the fluid in MOFs linearly increase as the temperature difference rises. It can be concluded that the thermal energy storage property of MOHCs is enhanced when the R161 is in a liquid state. Additionally, the more MOF-5 nanoparticles are added, the more thermal energy MOHCs can store. However, the MOHCs performed a negative enhancement of their thermal energy storage properties near the phase transition point of R161. This is because the enthalpy difference of the phase transition of R161 is large, which is more than the sum of the thermodynamic energy change of MOF-5 and the desorption heat of R161 in MOF-5 at this temperature range. Finally, the thermal energy storage property of MOHCs is enhanced when the temperature difference is over 140 K. Obviously, the sum of the thermodynamic energy change of MOF-5 and the desorption heat of R161 in MOF-5 is larger than the enthalpy difference of R161 when the temperature difference is over 140 K. This indicates that the MOHCs cannot be used for enhancing the thermal energy storage of a working fluid in certain conditions.

## 4. Conclusions

In this paper, molecular simulations (MD and GCMC methods), as well as thermodynamic calculation, are employed to investigate thermal energy storage by the adsorption of R161 in MOF-5. The R161/MOF-5 mixed MOHCs noticeably enhanced the thermal energy storage property of R161 in the liquid state. The thermal energy storage capacity of the studied MOHCs is less than that of pure R161 near the phase transition range. The MOHCs can be used for thermal energy storage when the sum of the thermodynamic energy change of an MOF and the desorption heat of the fluid in the MOF’s structure is larger than the enthalpy difference of the working fluid.

## Figures and Tables

**Table 1:** The desorption heat (kJ/mol) calculated by the Clausius–Clapeyron (C–C) equation and grand canonical Monte Carlo (GCMC).

C–C	GCMC_280K_	GCMC_300K_	GCMC_320K_	GCMC_340K_	GCMC_360K_	GCMC_380K_	GCMC_400K_	GCMC_420K_
41.74	48.58	48.34	48.10	47.62	47.51	47.16	47.01	46.85

## Nomenclature

*Δh_MOHCs_*	The enthalpy of MOHCs (kJ/kg)
*Δh_Fluid_*	The enthalpy of pure organic fluid (kJ/kg)
*Δh_desorption_*	The enthalpy of desorption (kJ/mol)
*T*	Temperature (K)
*C_p_*	The heat capacity of MOFs (kcal/(mol·K))
*q_st_*	The adsorption heat (kcal/(mol·K))
*x*	The mass fraction of MOF in MOHCs
*ρ*	Density (g/cm^3^)

## Appendix A. Adsorption Isotherms Fitted by the Langmuir Equation

Langmuir equation: M = a·b·p/(1 + a·p), where a and b are adsorption constants.

**Table A1:** The Langmuir equation fitting for R161 adsorption in MOF-5.

Temperature (K)	Constant a	Constant b
280	0.00690	21.027
300	0.00584	20.798
320	0.00564	20.567
340	0.00435	20.187
360	0.00364	19.670
380	0.00325	19.008
400	0.00299	18.340
420	0.00245	17.641
